# A Novel Risk Scoring Tool to Predict Saphenous Vein Graft Occlusion After Cardiac Artery Bypass Graft Surgery

**DOI:** 10.3389/fcvm.2021.670045

**Published:** 2021-08-12

**Authors:** Yujing Cheng, Xiaoteng Ma, Xiaoli Liu, Yingxin Zhao, Yan Sun, Dai Zhang, Qi Zhao, Yingkai Xu, Yujie Zhou

**Affiliations:** Beijing Key Laboratory of Precision Medicine of Coronary Atherosclerotic Disease, Department of Cardiology, Clinical Center for Coronary Heart Disease, Beijing Anzhen Hospital, Beijing Institute of Heart Lung and Blood Vessel Disease, Capital Medical University, Beijing, China

**Keywords:** coronary artery bypass grafting, coronary artery disease, graft occlusion, saphenous vein, risk factor

## Abstract

**Objectives:** Coronary artery bypass grafting (CABG) success is reduced by graft occlusion. Understanding factors associated with graft occlusion may improve patient outcomes. The aim of this study was to develop a predictive risk score for saphenous vein graft (SVG) occlusion after CABG.

**Methods:** This retrospective cohort study enrolled 3,716 CABG patients from January 2012 to March 2013. The development cohort included 2,477 patients and the validation cohort included 1,239 patients. The baseline clinical data at index CABG was analyzed for their independent impact on graft occlusion in our study using Cox proportional hazards regression. The predictive risk scoring tool was weighted by beta coefficients from the final model. Concordance (c)-statistics and comparison of the predicted and observed probabilities of predicted risk were used for discrimination and calibration.

**Results:** A total of 959 (25.8%) out of 3,716 patients developed at least one late SVG occlusion. Significant risk factors for occlusion were female sex [beta coefficients (β) = 0.52], diabetes (β = 0.21), smoking (currently) (β = 0.32), hyperuricemia (β = 0.22), dyslipidemia (β = 0.52), prior percutaneous coronary intervention (PCI) (β = 0.21), a rising number of SVG (β = 0.12) and lesion vessels (β = 0.45). On-pump surgery (β = −0.46) and the use of angiotensin-converting enzyme inhibitors (ACEI)/angiotensin receptor blockers (ARB) (β = −0.59) and calcium channel blockers (CCB) (β = −0.23) were protective factors. The risk scoring tool with 11 variables was developed from the derivation cohort, which delineated each patient into risk quartiles. The c-statistic for this model was 0.71 in the validation cohort.

**Conclusions:** An easy-to-use risk scoring tool which included female sex, diabetes, smoking, hyperuricemia, dyslipidemia, prior PCI, a rising number of SVG and lesion vessels, on-pump surgery, the use of ACEI/ ARB and CCB was developed and validated. The scoring tool accurately estimated the risk of late SVG occlusion after CABG (c-statistic = 0.71).

## Introduction

Coronary artery bypass grafting (CABG) surgery is a widely used treatment for complex coronary artery disease (CAD) that improves patient outcomes and prognosis (1). However, some patients experience myocardial ischemia recurrence after CABG. Studies have shown that the myocardial ischemic recurrence at 1 and 10 years after CABG are 17 and 63%, respectively (2, 3). The main cause of myocardial ischemic recurrence is graft failure (4). Saphenous vein is the most widely used vascular conduit for CABG (5); however, the estimated rate of occlusion is as high as 42% at a mean follow-up of 7.5 years (6). Graft occlusion is associated with worse quality of life and reduced long-term survival. Although the patency of saphenous vein graft (SVG) has been assessed in several studies (7). Late SVG occlusion, which defined as occlusion happened in the late phase (>12 months after grafting) (1, 8, 9) its mechanisms are different from early SVG occlusion. Late occlusion is due to development of atherosclerosis, which affects long-term clinical outcomes (10); the treatment is more difficult compared to early occlusion, the specific risk factors for late SVG occlusion remain unclear. Previous studies have suggested that sex (11), diabetes mellitus (12), and off-pump surgery (13) affect graft patency. However, there is no precise scoring model for late SVG occlusion.

The risk prediction model is an important tool for risk assessment. The risk scoring system is used to provide risk stratification, identify high-risk patients, control risk factors, and inform strategies to reduce mortality and improve quality of medical care. Previous SAFINOUS score is a simplified 12-variable risk scoring system that performs well in prediction of early SVG occlusion risk (14, 15). There is no standardized risk scoring system for late SVG occlusion. Therefore, it is of great clinical significance to establish a predictive risk scoring tool that accounts for specificity and accuracy. This study aimed to establish a risk scoring system that is suitable for CABG patients and evaluates the risk of late SVG occlusion.

## Methods

### Patients

Using the electronic medical system of Beijing Anzhen Hospital, we retrospectively identified 4,821 patients who underwent CABG surgery at our cardiac center between January 2012 and March 2013. Subsequently, we followed up with these patients between January 2017 and December 2017 to review and record their post-operative invasive angiography or coronary computed tomography angiography (CTA) results. Patients were excluded unless their medical records contained the following: (1) detailed pre-operative angiographic results; (2) saphenous vein used for the graft; (3) results of post-operative invasive angiography or CTA; and (4) detailed information on patency and occlusion of SVG. A total of 3,716 patients met the above criteria and were included in the final analysis (Supplementary Material 1). 2,568 patients were symptomatic, 592 patients underwent invasive angiography or CTA because of acute coronary syndrome. Type 2 diabetes mellitus was defined as (1) fasting blood glucose level higher than 7.0 mmol/L, (2) OGTT test positive, (3) Random blood glucose level higher than 11.1 mmol/L with typical symptoms of diabetes. Diabetes can be diagnosed if any one of the above is met. Smoker defined as smoking continuously or cumulatively for 6 months or more. The definition of dyslipidemia is elevated cholesterol, elevated low-density lipoprotein cholesterol, reduced high-density lipoprotein cholesterol, elevated triglycerides, or a combination of them. And patients with serum uric acid > 420 μmol/L in men, or >357 μmol/L in women were diagnosed as hyperuricemia. All data were retrieved from the electronic medical records system. Patient anonymity was ensured and this study was approved by the Institutional Review Board.

### Endpoints

If a patient had at least one SVG occlusion on follow-up invasive angiography or CTA, we regarded the patient to have reached the primary endpoint. The invasive angiography or CTA result was reviewed by two or more experienced cardiologists and a radiologist independently.

### Rationale for Risk Factor Selection

Risk factors selection was based on previous studies (7, 11, 12, 14, 16, 17) and clinical experience. Factors chosen were those easily measured and recorded. We recorded data on 38 relevant factors in this study (Table 1), and, after primary screening and Cox proportional hazards model analysis, 11 independent risk factors associated with late occlusion were selected.

**Table 1:** Patient characteristics.

	**Derivation cohort** ***N*** **= 2,477**	**Validation cohort** ***N*** **= 1,239**
Age (years)	59.73 ± 8.66	59.61 ± 8.88
Male gender, *n* (%)	1901 (76.7)	918 (74.1)
Obesity, *n* (%)	536 (21.6)	243 (19.6)
Diabetes, *n* (%)	1126 (45.5)	594 (47.9)
Hypertension, *n* (%)	1476 (59.6)	778 (62.8)
Hyperuricemia, *n* (%)	453 (18.3)	225 (18.2)
Dyslipidemia, *n* (%)	1429 (57.7)	708 (57.1)
Total cholesterol (mmol/L)	4.26 ± 1.20	4.27 + 1.24
High-density lipoprotein (mmol/L)	0.99 ± 0.24	0.98 ± 0.22
Low-density lipoprotein (mmol/L)	2.60 ± 0.96	2.62 ± 1.00
Triglyceride (mmol/L)	1.94 ± 1.48	1.90 ± 1.30
Platelet count (10^9^/L)	202.87 ± 58.07	206.78 ± 60.24
eGFR < 90, *n* (%)	1429 (57.7)	708 (57.1)
EF < 50, *n* (%)	203 (8.2)	125 (10.1)
ACS, *n* (%)	1059 (42.8)	562 (45.4)
Smoking, *n* (%)	1073 (43.3)	508 (41.0)
Drinking, *n* (%)	290 (11.7)	152 (12.3)
Prior stroke, *n* (%)	155 (6.3)	86 (6.9)
Prior myocardial infarction, *n* (%)	71 (2.9)	48 (3.9)
Prior PCI, *n* (%)	275 (11.1)	138 (11.1)
ACEI/ARB, *n* (%)	563 (22.7)	282 (22.8)
β blocker, *n* (%)	2163 (87.3)	1075 (86.8)
CCB, *n* (%)	666 (26.9)	337 (27.2)
Statin, *n* (%)	1663 (67.1)	819 (66.1)
Aspirin, *n* (%)	2316 (93.5)	1141 (92.7)
P2Y12 inhibitor, *n* (%)	1616 (65.2)	781 (63)
Diabetes therapy, *n* (%)	467 (18.9)	260 (21)
Complex coronary lesions, *n* (%)	1118 (45.1)	581 (46.9)
On-pump, *n* (%)	251 (10.1)	141 (11.4)
Valve surgery, *n* (%)	114 (4.6)	70 (5.6)
Reoperation, *n* (%)	39 (1.6)	14 (1.1)
Intra-aortic balloon pump, *n* (%)	17 (0.7)	4 (0.3)
Number of SVGs	3.08 ± 0.82	3.09 ± 0.85
Number of vessels with lesion	2.11 ± 0.84	2.10 ± 0.86
Left main, *n* (%)	542 (21.9)	279 (22.5)
LAD/Diagonal, *n* (%)	2033 (82.1)	992 (80.1)
LCX/OM, *n* (%)	1662 (67.1)	883 (71.3)
RCA, *n* (%)	1776 (71.7)	885 (71.4)
SVG occlusion, *n* (% patients)	643 (26.0)	316 (25.5)

*Values are given as mean + SD, or frequency n (percent). eGFR, estimated glomerular filtration rate; EF, ejection fraction; ACS, acute coronary syndrome; PCI, percutaneous coronary intervention; ACEI, angiotensin-converting enzyme inhibitors; ARB, angiotensin receptor blocker; CCB, calcium channel blocker; SVG, saphenous vein graft; LAD, left anterior descending branch; LCX, left circumflex branch; OM, obtuse marginal branch; RCA, right coronary artery*.

### Statistical Analysis and Predictive Model Development

Categorical variables are presented as number (percentage) and continuous variables as mean ± standard deviation. χ^2^ or Fisher's exact test was used for analysis of categorical variables. Unpaired student's *t*-test was used for analysis of normally distributed continuous variables. Time-to-event was calculated in months from the date of primary CABG to the date of post-operative invasive angiography or CTA. The construction of this predictive risk score was based on the method used in development of the Framingham risk score system (18). Development of the predictive risk score had three steps. First, to determine covariates that were independent risk factors for occlusion, we entered significant (*P* < 0.05) variables from the univariate analysis and/or those with clinical relevance into a multivariable Cox proportional hazards regression model using backward elimination with a critical *P* < 0.05. Second, to define the continuous risk factor, categories based on SVG number and number of vessels with lesions were used to determine a reference value (W_ij_). Categorical risk factors were modeled using sets of indicator variables. The referent risk factor profile (W_iREF_) was considered “not at risk” of SVG occlusion, and defined as category 1. We computed the distance from the category reference value to the referent value for each risk factor category by regression analysis using βi(W_ij_-W_iREF_). The β coefficient of the SVG number (continuous variable) was used as a reference standard and assigned one point, with the constant B equaling 0.12. Finally, the points associated with each category of risk factor were calculated via Points_ij_ = βi(W_ij_-W_iREF_)/B. The specific risk of each score was then calculated according to the previously described formula (18).

Model validation had two steps: discrimination and calibration. Discrimination of the predictive risk model was assessed using the c-index, which is equivalent to the area under the receiver operating characteristic (ROC) curve for binary dependent variables (19, 20), as an overall measure of model discrimination. Model calibration was assessed graphically using a calibration curve and observed vs. model-predicted late occlusion in risk groups. All statistical analyses were done with R (www.r-project.org; version 3.2.4) and SPSS (SPSS Inc., Chicago, Illinois, USA.; version 25.0). Graphs were generated using GraphPad Prism 8.3.0. All sections have been prepared according to the TRIPOD statement (21).

### Missing Data

Data for some variables were missing from our data set. Multiple imputation by chained equations (MICE) was used to impute missing values (22), which is superior to other methods (e.g., regression method, delete and mean method) and demonstrates stable performance.

## Results

### Study Population

During the follow up period, 959 (25.8%) patients developed at least one SVG occlusion. At the graft-level, 1,583 (18.8%) of the 8,422 grafts occluded within 5 years after primary CABG. This cohort was divided, after random sampling, into a derivation cohort (*n* = 2,477, 66.7%) and a validation cohort (*n* = 1,239, 33.3%). Baseline characteristics of patients in the derivation and validation cohorts were similar. The mean age of the derivation cohort was 59.73 ± 8.66 years and 76.7% were men. A total of 1,118 (60.3%) patients in the derivation cohort had complex coronary lesions, defined as left main lesion and/or triple-vessel lesion, 2,163 (87.3%) received β-blocker therapy, and 2,316 (93.5%) took aspirin after surgery (Table 1).

### Prediction Modeling for Late SVG Occlusion

In the multivariate COX proportional hazard model, 11 variables were identified as independent predictors for SVG occlusion: sex, diabetes mellitus, smoking, dyslipidemia, hyperuricemia, prior percutaneous coronary intervention (PCI), on-pump surgery, SVG number, lesion vessel number (including only vessels of the left anterior descending branch, circumflex artery, and right coronary artery), use of angiotensin-converting enzyme inhibitors (ACEI)/angiotensin receptor blockers (ARB), and use of calcium channel blockers (CCB; Table 2). Table 3 shows the β regression coefficient (βi), reference value (W_ij_), referent risk factor profile (W_iREF_), final point totals, and mean or proportion for each variable. Supplementary Material 2 shows the cumulative risk score associated with risk of late occlusion, with the theoretical range of point values between −9 and 24. Since there are few patients in the lower and upper ranges of the distribution, we shortened the risk table to avoid overstating the precision of the risk estimates. For example, a female patient (risk score = 4) who had diabetes mellitus (2) and hyperuricemia (2), a history of percutaneous coronary intervention (PCI; 2), and was a triple-vessel lesion patient (8), would have a final risk score of 18, with a predictive risk of 41% at 5 years of follow-up.

**Table 2:** Independent predictors for late SVG occlusion in the model.

**Risk factors**	**Regression coefficient**	**Hazards ratio (95% CI)**	**Mean or proportion**
SVG number	0.12	1.13 (1.03-1.24)	2.26
Number of vessels with lesion	0.45	1.57 (1.41-1.75)	2.11
Female	0.52	1.67 (1.37-2.04)	0.23
Dyslipidemia	0.52	1.68 (1.43-1.97)	0.58
Diabetes	0.21	1.23 (1.06-1.44)	0.46
Hyperuricemia	0.22	1.25 (1.03-1.51)	0.18
Smoking	0.32	1.38 (1.16-1.63)	0.47
ACEI/ABB	−0.59	0.56 (0.46-0.67)	0.23
Prior PCI	0.21	1.24 (1.01-1.54)	0.11
CCB	−0.23	0.80 (0.67-0.95)	0.27
On-pump	−0.46	0.63 (0.48-0.83)	0.10
Age	−0.002	0.99 (0.98-1.01)	0.71

*The final model was adjusted for age, obesity, hypertension, eGFR < 90, aspirin, P2Y12 inhibitor, complex coronary lesions. CI, Confidence interval; SVG, saphenous vein graft; ACEI, angiotensin-converting enzyme inhibitors; ARB, angiotensin receptor blocker; CCB, calcium channel blocker; PCI, percutaneous coronary intervention; eGFR, estimated glomerular filtration rate*.

**Table 3:** Predictors from Cox proportional hazard model used in the construction of the SVG occlusion score.

**Risk factors**	**Categories**	**Reference value (Wij)**	**βi**	**βi (Wij –Wiref)**	**Point**
SVG number	1	1 = W1ref	0.12	0	0
	2	2		0.12	1
	3	3		0.24	2
	4	4		0.36	3
	>4	5		0.48	4
Number of vessels with lesion	0	0	0.45	−0.45	−4
	1	1 = W2ref		0	0
	2	2		0.45	4
	3	3		1.11	8
Female	No	0 = W4ref	0.52	0	0
	Yes	1		0.52	4
Dyslipidemia	No	0 = W3ref	0.52	0	0
	Yes	1		0.52	4
Diabetes	No	0 = W5ref	0.21	0	0
	Yes	1		0.21	2
Hyperuricemia	No	0 = W7ref	0.22	0	0
	Yes	1		0.222	2
Smoking	No	0 = W6ref	0.32	0	0
	Yes	1		0.32	3
ACEI/ARB	No	0 = W8ref	−0.59	0	0
	Yes	1		−0.59	−5
Prior PCI	No	0 = W9ref	0.21	0	0
	Yes	1		0.21	2
CCB	No	0 = W10ref	−0.23	0	0
	Yes	1		−0.23	−2
On-pump	No	0 = W11ref	−0.46	0	0
	Yes	1		−0.52	−4

*SVG, saphenous vein graft; ACEI, angiotensin-converting enzyme inhibitors; ARB, angiotensin receptor blocker; CCB, calcium channel blocker; PCI, percutaneous coronary intervention*.

### Summary Measures of Calibration and Discrimination

Discrimination of the predictive risk model was assessed using the c-index. The final predictive model had good performance for prediction of late SVG occlusion in the derivation cohort (c-index = 0.69; 95% CI, 0.67-0.71). The predictive model also had good performance in the validation cohort (c-index = 0.73; 95% CI, 0.77-0.80).

**Figure 1:**
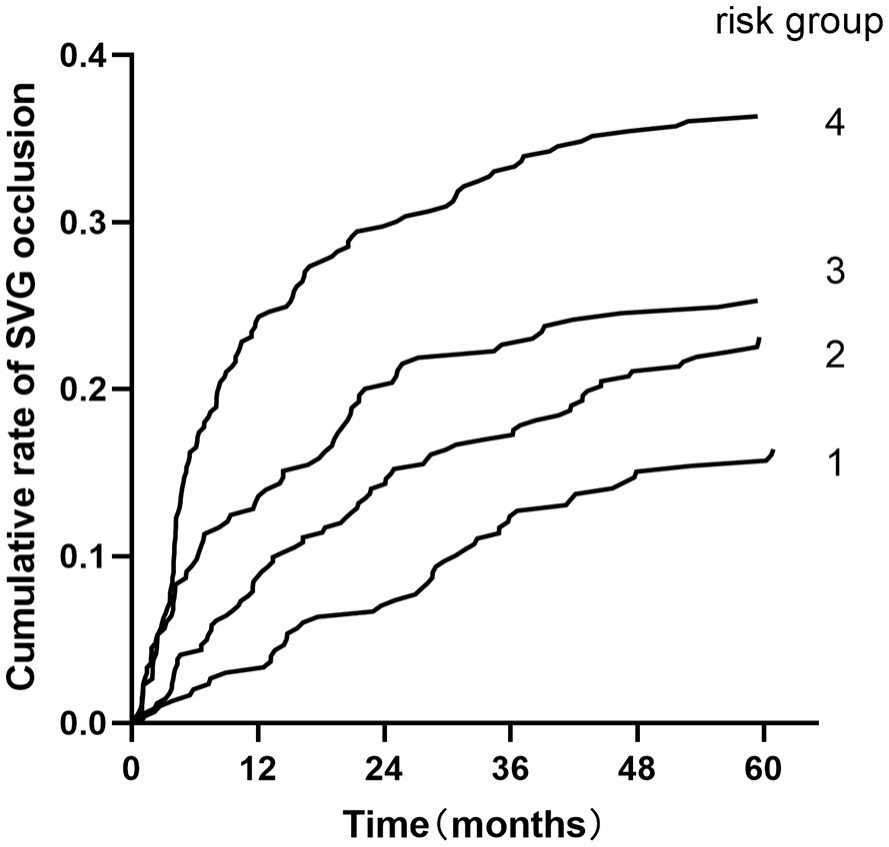
Cumulative rate of SVG occlusion for patients classified into four groups based on the developed risk score. Risk groups 1-4 represent risk scores ≤ 5, 6-10, 11-13, and ≥14. SVG, saphenous vein graft.

**Figure 2:**
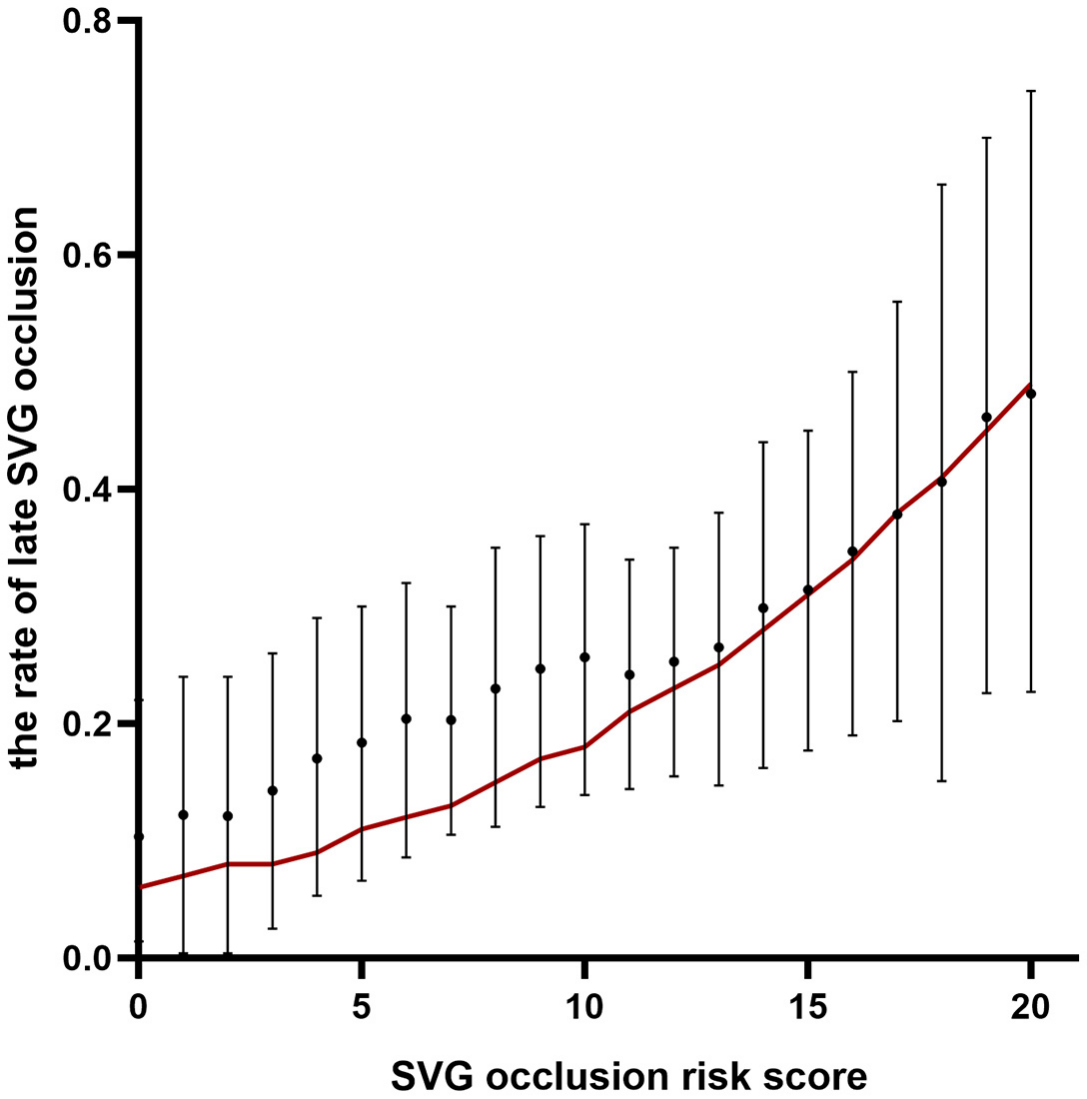
Observed rate of occlusion with 95% confidence interval vs. model-predicted risk of occlusion in groups based on the developed risk score. SVG, saphenous vein graft.

**Figure 3:**
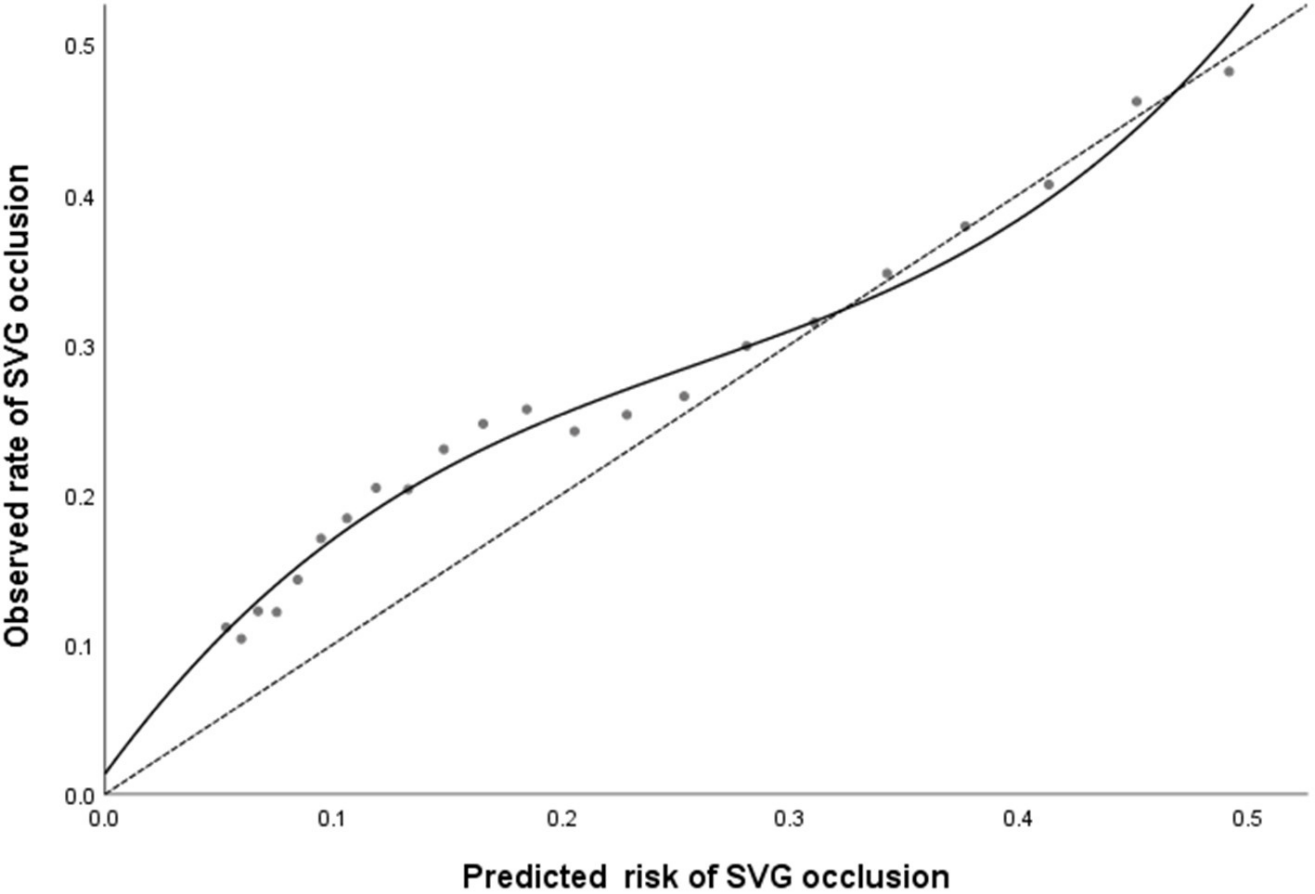
The calibration plot presents the predicted risk against the observed proportion of late SVG occlusion for 21 groups based on the calculated SVG occlusion risk score. A locally weighted regression line is plotted to show the general trend. The dashed line is the line of reference shows the ideal calibration line. SVG, saphenous vein graft.

Patients were classified into four groups representing the quartiles of risk. The first to fourth quartiles contained 299, 342, 265, and 333 patients, respectively, and corresponded to a score of ≤ 5, 6-10, 11-13, and ≥14, respectively. Discrimination was good, as is shown in the plot of cumulative rate of late SVG occlusion for patients classified into each of the four risk groups (Figure 1). The observed vs. predicted rates of late SVG occlusion in the first to fourth quartiles is shown in Supplementary Material 3. The exact occlusion rates for each risk group (observed vs. predicted) were 16.4 vs. 7.8%, 23.1 vs. 15.3%, 25.2 vs. 23.9%, and 36.3 vs. 36.2%, respectively. The difference between the observed and model-predicted late SVG occlusion risks was 8.6, 7.8, 1.3, and 0.1% for risk groups 1–4, respectively. There was an underprediction of SVG occlusion in the two lower risk groups, and a relatively precise prediction in the two higher risk groups. A modest underestimation in the lower probability range and a relatively precise estimation in the higher probability range of late SVG occlusion were also evident from calibration plots (Figures 2, 3). Figure 2 presents the observed late SVG occlusion rate vs. model-predicted risk in groups based on the SVG occlusion risk score. In Figure 3, the calibration plot presents the mean predicted risk of late SVG occlusion against the observed proportion of late SVG occlusion for 21 groups based on the calculated SVG occlusion risk score. Visually, it appeared to be a good calibration of both the predictive risk model and observed proportion across every risk score from 0-20.

## Discussion

We developed a predictive risk score for saphenous vein graft SVG occlusion after CABG. We found that the model discrimination was good; in other words, the risk score was reliable in correctly classifying patients via risk stratification. However, the calibration was not ideal, which caused a modest underestimation of late SVG occlusion for low risk patients, while demonstrating a relatively precise predictive performance for high risk patients. There are a total of 11 independent predictive factors in the scoring system that leads to calculation of a CABG patient's personalized risk of developing late SVG occlusion. The risk score developed in this study could guide treatment strategy by focusing on the likelihood of late SVG occlusion in CABG patient with high-risk factors, such as hyperuricemia and the use of ACEI/ARB or CCB, which are not currently addressed in treatment recommendations. This risk score has significant implications for CABG patients, as those with higher risk scores should be managed with greater vigilance and intensive treatment to effectively mitigate cardiovascular risk factors.

CABG is one of the most effective revascularization strategies for CAD, especially for patients with multivessel diseases, and has been shown to reduce mortality and improve quality of life (23). However, SVG occlusion has an adverse impact on the prognosis of patients and increases the economic burden of health care systems (6). SVG occlusion can be classified into two types: early and late. Early SVG occlusion is primarily attributed to a technical failure that results in graft thrombosis and hyperplasia as the SVG is arterialized. Late SVG occlusion is primarily due to generalized neointimal hyperplasia and atherosclerosis, which occurs over the injured endothelium (1). The risk factors associated with late SVG occlusion have been evaluated in some studies (7, 13, 17, 24, 25), but a widely accepted prediction model for late SVG occlusion had not been previously developed. We have designed and validated a prediction model for late SVG occlusion by using cohort data from a high-volume cardiac center. Previous studies have shown specific risk factors from patient-related, graft-related, and surgery-related perspectives. Female sex is an independent risk factor of SVG occlusion in early vein graft failure (11, 12, 16), possibly due to smaller target vessel diameter of female patients. Cardiovascular risk factors like diabetes, smoking, dyslipidemia, and a history of PCI have also been identified as risk factors of late SVG occlusion (7, 17). Uric acid level had never been considered relevant to SVG occlusion, however, clinical practice experience and prior research indicate that hyperuricemia leads to kidney injury (26), and chronic kidney disease has been reported as a risk factor for vein graft disease (24, 27). Off-pump surgery for CABG without cardioplegia has been associated with lower graft patency rates compared with on-pump surgery. Additionally, the coagulopathy and platelet dysfunction induced by cardiopulmonary bypass can affect SVG patency (25, 28). From the graft-related and surgery-related perspectives, any use of SVGs is independently associated with reduced survival after coronary artery bypass surgery (29), which is consistent with the risk factors we have derived. Most patients with diseases with multiple lesions have diffuse lesions, suggesting that the condition of the graft may be poor; these patients have a higher rate of late SVG occlusion. As for medications, the use of ACEI/ARB and CCB are both protective factors for SVG occlusion, which may be related to the dilation of blood vessels, antispasmodics, and increased graft diameter. Furthermore, the effects of antihypertensive medications may contribute to reduced risk of SVG hyperplasia, which has been demonstrated in a study on early SVG occlusion (30).

A variety of cardiac surgery risk prediction models have been established, including the American Thoracic Surgery (STS) score (31), the American College of Cardiology/American Heart Association (ACC/AHA) score (32), the European EuroSCORE (33), and its modified version, the EuroSCORE II (34). These prediction models were primarily used for evaluating perioperative risk. For SVG occlusion, the SAFINOUS score (14) is a model for early SVG occlusion, with risk factors that include sex, diabetes, dyslipidemia, active smoking, and SVG number, which are consist with the risk factors identified in our study. A study by Sabik et al. also found that female sex and diabetes are risk factors for graft occlusion (16). Moreno et al., Wezel et al., and Yazdani et al. found that atherosclerosis and plaque rupture are the main causes of late vein graft failure. The formation of atheromatous plaques is promoted by predisposing factors for atherosclerosis (e.g., high blood pressure, diabetes, smoking), and damage to the vein wall is induced by highly proliferative smooth muscle cells and expression and secretion of pro-inflammatory cytokines (10, 35–37). These findings are consistent with some of our results. Domanski et al. focused on prognostic factors for atherosclerosis progression in saphenous vein grafts, hypothesized that dyslipidemia, prior myocardial infarction, female sex and current smoker status were associated with acceleration of the atherosclerosis progression in saphenous vein grafts, thereby leading to late SVG occlusion (38).

Our study is the first large-scale comprehensive cohort–based development of a predictive model for late SVG occlusion that could be used for risk stratification of CABG patients. The risk score system could inform clinical decision-making through calculation of individual risk for late SVG occlusion. Assessment of the SVG risk score could improve surgical strategy and help in the development of personalized post-operative treatment plans. Proactive risk assessment and associated treatment may also be particularly cost-effective by reducing SVG occlusion and cardiovascular events in patients after CABG.

We recognize that our study has limitations. First, this research is a retrospective investigation. Although we continuously enrolled patients who underwent CABG, a significant number of patients did not receive invasive angiography or CTA results during the follow-up time, and the CAD duration was not recorded, which may result in selection bias. The time of post-operative invasive angiography or CTA depends on many factors. The most important factors are the patient's symptoms and the personal decision of the doctors providing outpatient services. Although this may affect the observation of the SVG occlusion, it is the best representation of the current context of clinical practice. There was also data missing from our database. Although we used MICE to impute missing values, bias is evitable. In addition, the absence of some variables, such as target vessel diameter, graft mean flow, and some surgical related items, leads to a risk of confounding. Finally, our database was split into two groups randomly: one to develop a prediction model, and one to evaluate the predictive performance of the model. This design led to lack of power during model development (21, 39–41) and validation. The development and validation cohort has a total of 3,716 people, the sample size is relatively small, and it is a single-center study. The model includes more variables (11 variables) relatively, which may cause potential bias. Future validation studies carried out at different medical centers with different surgical strategies and external validation by other investigators would be welcome.

## Conclusions

We developed a comprehensive prediction model which included 11 variables (male sex, diabetes, smoking, hyperuricemia, dyslipidemia, prior PCI, a rising number of lesion vessels, and SVG) for late SVG occlusion based on a large cohort of CABG patients. The risk score had a good capacity for risk stratification, with a modest underestimation of SVG occlusion for low risk patients and a relatively precise prediction among high risk patients. The scoring tool is an 11-variable risk scoring system that can independently predict late SVG occlusion and be used to improve clinical management by identifying high-risk patients and informing surgical strategy.

## Data Availability Statement

The data analyzed in this study is subject to the following licenses/restrictions: the policy of our hospital. Requests to access these datasets should be directed to 13426481193@163.com.

## Author Contributions

YZho and XL contributed to drafting the article or revising it critically for important intellectual content. YC and XM made the primary contributions to conception and design, and should be considered co-first authors. All authors contributed to acquisition of data, or substantial analysis and interpretation of data, and final approval of the version to be published.

## Conflict of Interest

The authors declare that the research was conducted in the absence of any commercial or financial relationships that could be construed as a potential conflict of interest.

## Publisher's Note

All claims expressed in this article are solely those of the authors and do not necessarily represent those of their affiliated organizations, or those of the publisher, the editors and the reviewers. Any product that may be evaluated in this article, or claim that may be made by its manufacturer, is not guaranteed or endorsed by the publisher.
