# Supplementary material for: A Novel Risk Scoring Tool to Predict Saphenous Vein Graft Occlusion After Cardiac Artery Bypass Graft Surgery
Source: Front Cardiovasc Med. 2021 Aug 12;8:670045. doi: 10.3389/fcvm.2021.670045 (PMC8387700; doi:10.3389/fcvm.2021.670045)
Supplement: Supplementary file 1 [file Data_Sheet_1.docx]

**Supplementary 2. Cumulative risk score and associated risk of late SVG occlusion**

| score | Risk of late SVG occlusion |
| --- | --- |
| -1 or less | ≤0.05 |
| 0 | 0.06 |
| 1 | 0.07 |
| 2 | 0.08 |
| 3 | 0.08 |
| 4 | 0.09 |
| 5 | 0.11 |
| 6 | 0.12 |
| 7 | 0.13 |
| 8 | 0.15 |
| 9 | 0.17 |
| 10 | 0.18 |
| 11 | 0.21 |
| 12 | 0.23 |
| 13 | 0.25 |
| 14 | 0.28 |
| 15 | 0.31 |
| 16 | 0.34 |
| 17 | 0.38 |
| 18 | 0.41 |
| 19 | 0.45 |
| 20 | 0.49 |
| 21 or more | ≥0.53 |

Suppl.1 Flow of Study Participants in this Study. CABG: Coronary artery bypass grafting; CTA: coronary computed tomography angiography; SVG: saphenous vein graft

Suppl.3 Observed vs. model-predicted late SVG occlusion in four risk groups. Risk groups 1-4 represent risk scores ≤5, 6-10, 11-13, and ≥14. SVG: saphenous vein graft
