# Supplementary figures and images for: A Novel Risk Scoring Tool to Predict Saphenous Vein Graft Occlusion After Cardiac Artery Bypass Graft Surgery
Source: Front Cardiovasc Med. 2021 Aug 12;8:670045. doi: 10.3389/fcvm.2021.670045 (PMC8387700; doi:10.3389/fcvm.2021.670045)

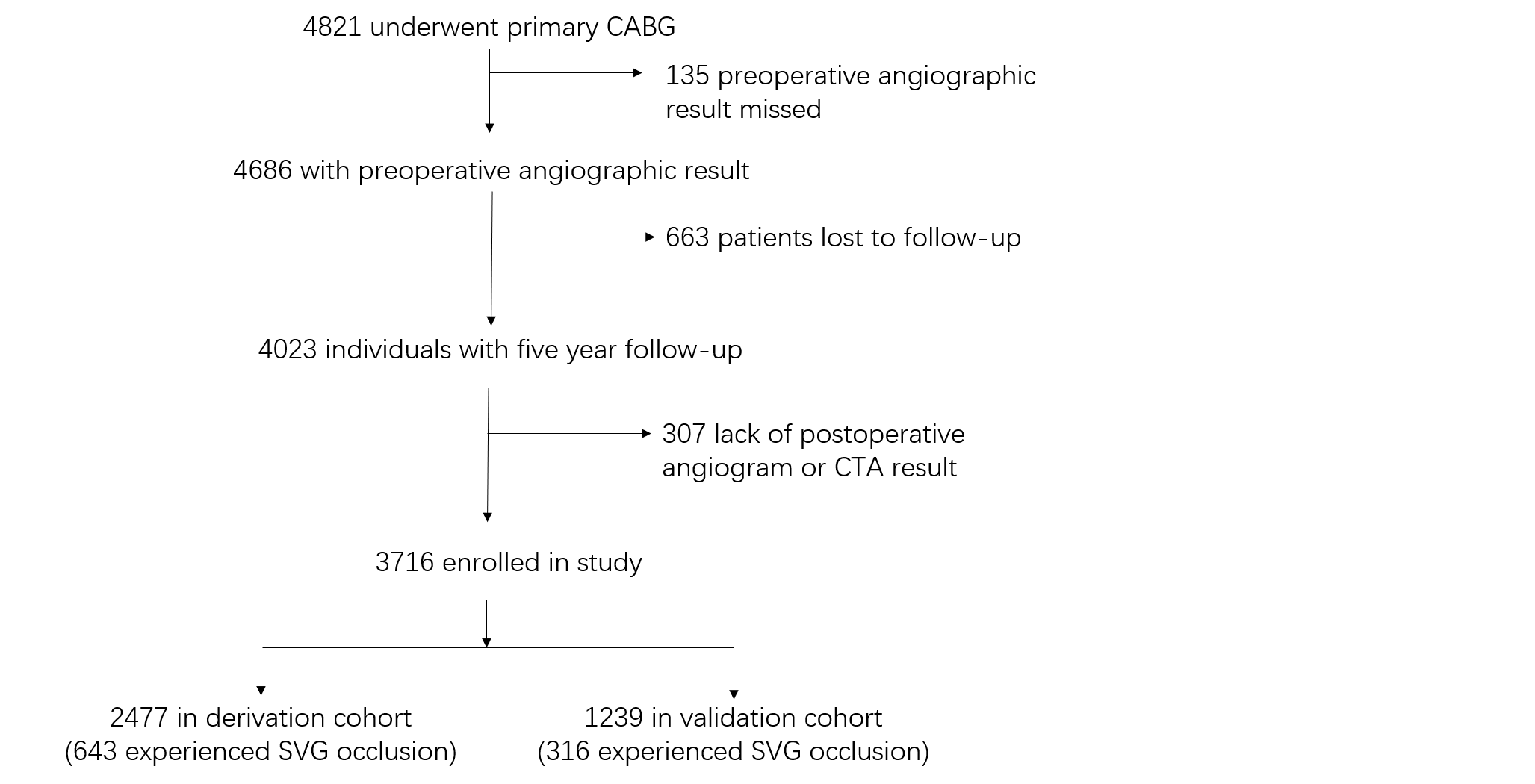

Supplement: Supplementary file 2 [file Image_1.TIF]

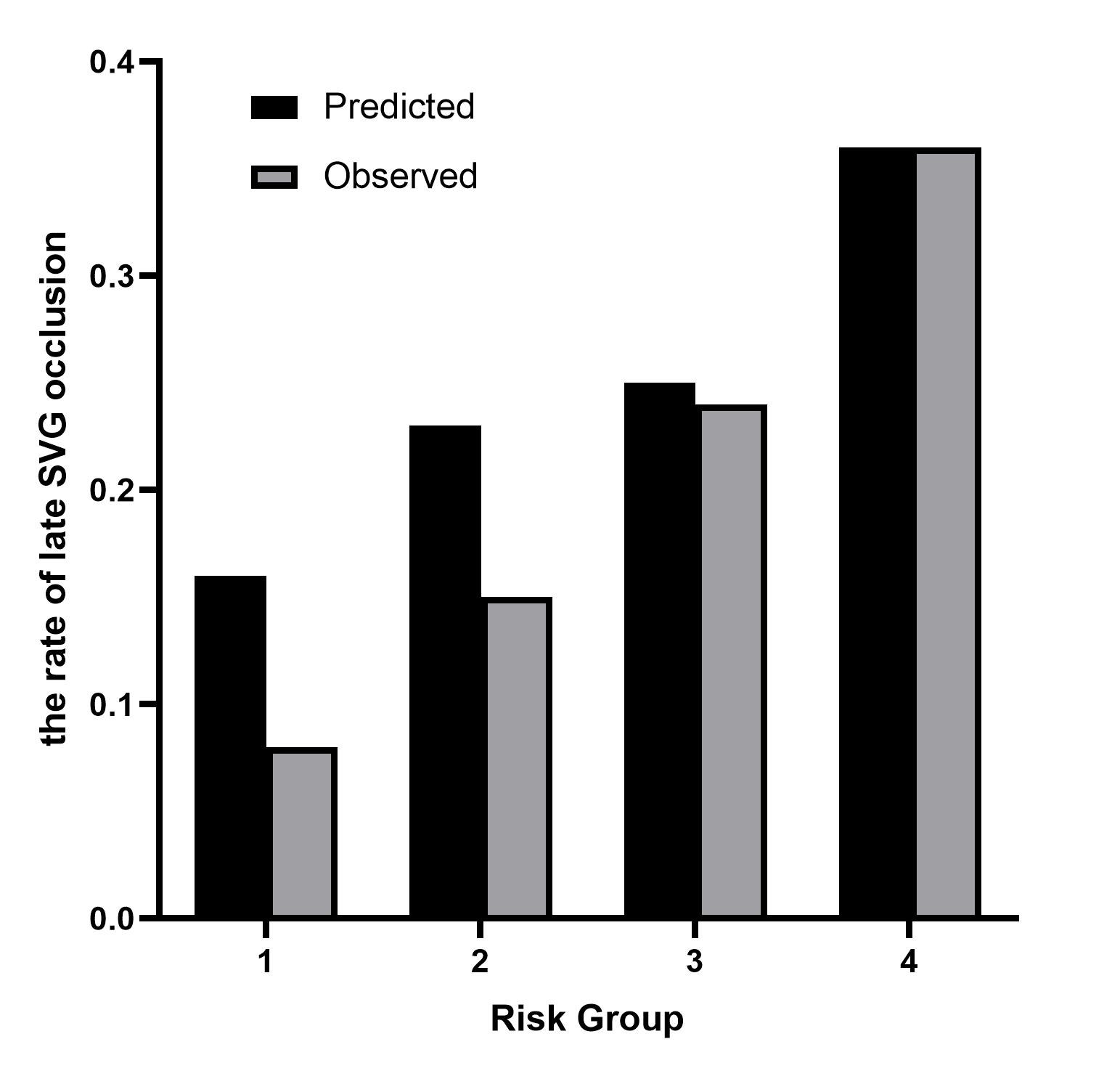

Supplement: Supplementary file 3 [file Image_2.TIF]
